# The outcome of Infliximab induction in patients with severe ulcerative colitis may be related to intestinal microbiota

**DOI:** 10.3389/fmicb.2026.1779143

**Published:** 2026-05-21

**Authors:** Jie Chen, Qian Zhou, Xuemin Cao, Shoulu Jin, Yingying Wu, Tianqi Ren, Chenjing Xu, Han Chen, Hongjie Zhang, Yue Zhang, Jun Liu

**Affiliations:** 1 Department of Gastroenterology, Northern Jiangsu People’s Hospital, Yangzhou, China; 2 The Affiliated Subei People’s Hospital of Yangzhou University, Yangzhou, China; 3 Department of Gastroenterology, Nanjing First Hospital, Nanjing, China; 4 Department of Gastroenterology, The First Affiliated Hospital with Nanjing Medical University, Nanjing, China

**Keywords:** efficacy prediction, gut microbiota, infliximab, ulcerative colitis, untargeted metabolomics

## Abstract

**Background and objective:**

Although infliximab (IFX) is endorsed for induction therapy in severe ulcerative colitis (SUC), its therapeutic response remains heterogeneous. We conducted a comprehensive analysis of fecal and mucosal microbiota, coupled with targeted metabolomics, to delineate microbial and metabolic signatures predictive of IFX induction efficacy and to explore mechanistic pathways underlying differential treatment responses.

**Methods:**

This study was a prospective cohort study (clinical study registration number: ChiCTR2300071816). It enrolled adult patients aged ≥ 18 years who were first diagnosed with SUC at the First Affiliated Hospital of Nanjing Medical University and Northern Jiangsu People’s Hospital between February 2022 and December 2023. None of these patients had received any medication for UC, including antibiotics. Clinical data, fecal samples, and rectal mucosal samples were collected for analysis. High-throughput sequencing of the 16S rRNA gene and non-targeted metabolomics analysis were performed on both fecal and rectal mucosal samples. All patients underwent IFX (5 mg/kg) induced remission therapy at weeks 0, 2, and 6. Based on their clinical response at week 14, they were categorized into two groups: response and unresponse. Significant differences in bacterial composition between the two groups were identified by screening fecal and mucosal samples. The gut microbiota of feces and intestinal mucosa were combined with clinical data to create four prediction models and conduct comparisons. Furthermore, different metabolites from fecal and mucosal samples of the two groups were screened and compared with KEGG and PubChem databases to identify metabolic pathways associated with the efficacy of IFX-induced therapy.

**Results:**

Compared with non-responders, IFX responders harbor distinct gut microbiota. Clinical indices alone poorly forecast induction response (AUC 0.6429). Augmenting clinical variables with fecal or mucosal microbiota improves prediction to AUC 0.795 and 0.900, respectively; combining both compartments further elevates performance to AUC 0.964, indicating that integrated microbiome profiling is essential for optimal IFX response prediction. IFX responders and non-responders differ metabolically, with more discriminatory features in mucosa than feces. The most enriched differential pathways in feces included nicotinate and nicotinamide metabolism, butyrate metabolism, biosynthesis of valine, leucine, and isoleucine, pantothenate and coenzyme A biosynthesis, histidine metabolism, and alanine, aspartate, and glutamate metabolism. In mucosa, the most enriched differential pathways included alanine, aspartate, and glutamate metabolism, sphingolipid metabolism, ascorbate and aldarate metabolism, tryptophan metabolism, and D-glutamine and D-glutamate metabolism. The common pathway enriched in both feces and mucosa was alanine, aspartate, and glutamate metabolism.

**Conclusion:**

The intestinal microbiota may be a predictive factor for IFX induction outcome in patients with SUC. The metabolic pathway of alanine, aspartate, and glutamate may be associated with the 14-week clinical response to IFX treatment in SUC.

## 1 Introduction

The 2020 AGA Clinical Practice Guidelines for the Management of Moderate to Severe Ulcerative Colitis(UC)recommend the use of infliximab (IFX), adalimumab, golimumab, vedolizumab, tofacitinib, or ustekinumab for the treatment of moderate-to-severe UC in the outpatient setting (Feuerstein et al., 2020). However, biologic agents exhibit a plateau in efficacy, with research indicating that the short-term (8-week) clinical response rate to infliximab (IFX) in patients with moderate-to-severe UC ranges from 65.5 to 69.4%, while clinical remission rates are between 33.9 and 39% (Rutgeerts et al., 2005). Moreover, the use of biologics can also lead to primary non-response, characterized by a failure of patients to respond to induction therapy, with an incidence ranging from 20 to 40% (Swaminath et al., 2009). The early identification of patients who demonstrate a lack of response to biologic therapies, combined with the timely modification of their treatment regimens, can significantly improve therapeutic effectiveness, reduce potential risks, and lower treatment costs.

In recent years, there has been an increasing focus on research aimed at leveraging the composition of the gut microbiome and its associated metabolic changes to predict the efficacy of anti-tumor necrosis factor (TNF) therapies for inflammatory bowel disease (IBD), particularly in relation to Crohn’s disease (CD). Zhou et al. (2018) demonstrated that Clostridiales can serve as a reliable predictor of response to IFX treatment in patients with CD. Aden et al. (2019) discovered that the metabolic exchange in fecal samples from patients with inflammatory bowel disease was significantly diminished at baseline, which correlated with subsequent clinical remission. Fecal metabolomics analysis further confirmed a significant association between the predicted levels of substrates involved in butyrate synthesis (such as ethanol or acetaldehyde) and clinical remission following IFX treatment. Wang et al. (2021) discovered that IFX may exert its effects, in part, by promoting the proliferation of bacteria that produce short-chain fatty acids and bile acids. This process contributes to the suppression of inflammation and the restoration of bile acid metabolism. Ventin-Holmberg et al. (2021) discovered that individuals who did not respond to IFX treatment exhibited a reduced abundance of short-chain fatty acid-producing bacteria, particularly within the Clostridia class. In contrast, these non-responders displayed an increased presence of pro-inflammatory bacteria and fungi, such as Candida. These findings suggest that the composition of gut microbiota may serve as a predictive factor for the response to IFX in patients with CD and UC (Ventin-Holmberg et al., 2021).

Based on this premise, the present study aims to analyze the gut microbiota and metabolites in both fecal samples and intestinal mucosal tissues of patients with severe ulcerative colitis (SUC) who either responded or did not respond to IFX induction therapy. The objective is to identify microbiomic characteristics that can predict IFX response and to integrate these findings into a multi-parametric predictive model for IFX efficacy. This approach seeks to assist clinicians in making timely adjustments to treatment plans and optimizing therapeutic strategies.

## 2 Materials and methods

This study was a multicenter prospective cohort study. According to the “Consensus on Diagnosis and Treatment of Inflammatory Bowel Disease (2018, Beijing)” (Inflammatory Bowel Disease Group et al., 2021), a total of 50 newly diagnosed patients with SUC—who had not previously received treatment with antibiotics, mesalazine, corticosteroids, biologics, or immunosuppressive agents—were enrolled from January 2022 to December 2023 at The First Affiliated Hospital of Nanjing Medical University and Northern Jiangsu People’s Hospital. All patients were treated with IFX as first-line therapy. Clinical data, fecal samples, and intestinal mucosal samples were collected prior to treatment. The primary observation endpoint was the clinical response assessed at week 14, marking the conclusion of the induction therapy. Patients were categorized into two groups based on their clinical response: response (*n* = 36) and unresponse (*n* = 14). Fecal and mucosal samples were subjected to 16S rRNA high-throughput sequencing. The alpha and beta diversity of fecal and mucosal microbiota were compared between the two groups, and Linear Discriminant Analysis Effect Size (LEfSe) was used to identify significantly different bacteria. The relative abundance of significantly different bacteria in the response and unresponse groups was utilized as microbiota variables, while clinical indicators reflecting the severity of UC disease including disease extent, endoscopic index of severity prior to treatment, C-reactive protein (CRP), platelets (PLT), erythrocyte sedimentation rate (ESR), albumin (ALB), and hemoglobin (Hb) were employed as clinical variables. These variables were incorporated into a Random Forest (RF) model to construct four models: One using only clinical data, another combining clinical data with fecal microbiota, a third combining clinical data with mucosal microbiota, and finally one combining clinical data with both fecal and mucosal microbiota. The best predictive model was determined by comparing the area under the operating characteristic curve (AUC) of these models. LC-MS/MS mass spectrometry was employed for the analysis of metabolic profiles in fecal and rectal mucosal samples from both groups. Differential metabolites were identified through univariate and multivariate statistical analyses. Subsequently, these differential metabolites were annotated against authoritative metabolite databases such as KEGG and PubChem.

### 2.1 Total bacterial genomic DNA extraction

Mucosal bacterial DNA was extracted with the TIANamp Bacteria DNA Kit (DP302-02; Tiangen Biotech), and fecal DNA with the Magbead Stool Genomic DNA Kit (AU46111-96; Bioteke), respectively, following the manufacturers’ protocols. DNA concentrations were determined using a NanoDrop ND- 1000 (NanoDrop, Wilmington, DE, United States), and the remaining samples were preserved at –80°C prior to the performance of PCR.

### 2.2 PCR amplification and Illumina sequencing

We used bar-coded primers 515F (5’-GTGYCAGCMGCCGCGGTAA-3’) and V4-806R (5’- GGACTACHVGGGTWTCTAAT-3’) to amplify the bacterial 16S rRNA V4 fragments. The PCR cycle conditions were as follows: initial denaturation at 98°C for 30 s; 32 cycles of 98°C for 10 s, 54°C for 30 s, and 72°C for 44 s; and final extension at 72°C for 10 min. Each 25 μL reaction mixture consisted of 12.5 μLTaKaRa Premix Taq (D331A, version 2.0; TaKaRa Biotech, Dalian, China), 5 μL dsDNA (Invitrogen, Life technologies, United States), 2.5 μL bar-coded primer V4-515F, 2.5 μL primer V4-806R, and 2.5 double-distilled water (ddH2O). PCR products were gel purified using AMPure XT beads (Beckman, United States) and sequenced using the 250-bp paired-ended strategy on an Illumina NovaSeq at Shanghai Biotree Biotech.

### 2.3 Bioinformatics analysis

All sample sets were sequenced on the Illumina NovaSeq platform. We obtained a total of 7,118,505 raw reads, with an average of 71,185.05 ± 11,827.39 reads per sample, ranging from 41,233 to 80,615. Raw data processing was performed as follows: Sequencing primers were removed using cutadapt (v1.9). Paired-end reads were merged using FLASH (v1.2.8). Quality filtering was performed using fqtrim (v0.94) to remove low-quality reads (quality scores < 20), short reads (< 100 bp), and reads containing more than 5% “N” bases. Chimeric sequences were identified and removed using Vsearch (v2.3.4). Subsequently, DADA2 was applied for denoising and generating amplicon sequence variants (ASVs). Taxonomic annotation was performed using the QIIME2 feature-classifier plugin (2019.7) against the SILVA database (release 138). To calculate alpha and beta diversity, the data were rarefied to a sequencing depth of 41,233 reads per sample. Alpha diversity (Chao and Shannon indices) and beta diversity (Bray-Curtis distance matrices) were calculated using QIIME2. Differentially abundant bacteria were identified using LEfSe (LDA ≥ 3.0, *p* < 0.05). Visualization was performed using R (version 3.5.2).

All 16s sequences were concatenated and subjected to a single closed-reference OTU-picking step. RF classifiers were then built on the resulting OTU abundance matrix using the caret package (v 4.6–14) in R. We employed a Recursive Feature Elimination with Cross-Validation (RFECV) framework based on RF classifiers. Specifically, the methodology utilized 10-fold stratified cross-validation as the core resampling strategy. Within each training fold, a RF model was constructed to compute variable importance scores, based on which features were progressively eliminated following a logarithmic scale (step = 0.6). Subsequent models were trained on these nested feature subsets, and classification errors were evaluated on the respective validation folds. This entire procedure was repeated for 5 iterations to ensure robustness. The mean and standard deviation of cross-validation errors corresponding to different feature set sizes were compared, with the subset yielding the minimum error designated as the optimal feature set. The final RF predictive model was then constructed using these selected features. Model performance was assessed by generating Receiver Operating Characteristic (ROC) curves and quantifying the AUC values. Feature importance was gauged by the increase in prediction error observed when a given OTU’s values were randomly permuted while all others were held constant. Functional profiles in terms of KEGG Orthologs (KOs) were inferred from the 16S data with PICRUSt, and the predicted KO abundances were subsequently collapsed to KEGG Pathway Level 3 to obtain higher-level functional categories.

### 2.4 Non-targeted metabolomics analysis

The mass spectrometry analysis of fecal and mucosal samples was conducted using LC-MS/MS. The samples were qualitatively and quantitatively analyzed using the KEGG compound database, followed by cluster heatmap analysis and principal component analysis (PCA) to compare the differences in metabolites among each group. Due to the high-dimensional and small-sample characteristics of metabolomics data and the potential influence of related variables on the distribution of differential variables, we adopted the orthogonal projections to latent structures-discriminant analysis (OPLS-DA) statistical method to further analyze the results of PCA. This method can effectively screen out orthogonal variables unrelated to classification and analyze both non-orthogonal and orthogonal variables, thereby accurately determining the differences in metabolites between groups and their correlation with the experimental group. The qualitative and quantitative results of metabolites were screened out through univariate and multivariate statistical analyses. The screening criteria were defined as: 1. *t*-test *P* < 0.05; 2. The variable projection importance (Variable Importance in the Projection, VIP) of the first principal component in OPLS-DA analysis > 1. To further precisely screen out the differential metabolites related to clinical parameters required by the experiment, all samples were divided into test sets and validation sets at a 1:1 ratio, and the common differential metabolites between the two were sought.

Pathway enrichment analysis of differential metabolites was performed using commercial databases including KEGG^1^ and MetaboAnalyst^2^. Metabolite annotations (compound IDs and peak intensities) were mapped to the Homo sapiens pathway database in KEGG to identify significantly enriched metabolic pathways, and MetaboAnalyst was used for pathway visualization and statistical analysis (Kanehisa et al., 2023). The results of the metabolic pathway analysis were visualized by bubble charts to facilitate the identification of key metabolic pathways influenced by differential metabolites.

This study received ethical approval from the Ethics Committees of the First Affiliated Hospital with Nanjing Medical University and Northern Jiangsu People’s Hospital (approval numbers: 2022-SR-530, 2023Ky043). Additionally, it was registered with the Chinese Clinical Trial Center (registration number: ChiCTR2300071816). Informed consent forms were signed by all participants.

## 3 Statistical analysis

All statistical analyses were conducted using SPSS 27.0 software (SPSS, Chicago, IL, United States). Quantitative data are expressed as mean ± standard deviation (SD) or as median and interquartile ranges (IQR). Qualitative data are presented as frequency or percentage. When continuous data followed a normal distribution, *t*-tests were used for comparisons between two groups, and ANOVA was used for comparisons among three or more groups. For data that did not follow a normal distribution, *U*-tests were applied for comparisons between two groups, and the Kruskal-Wallis H test was used for comparisons among three or more groups. All significance tests were two-tailed, and a *p* < 0.05 considered statistically significant.

## 4 Results

### 4.1 General data

The 50 enrolled patients with SUC all received IFX as first-line therapy. Based on their clinical response at week 14, the patients were categorized into groups of clinical responders and non-responders. The gender, age, baseline disease extent, Endoscopic index of severity, CRP, PLT, ESR, Hb, and ALB were summarized for both groups. The severity of endoscopic findings was evaluated using the Ulcerative Colitis Endoscopic Index of Severity (UCEIS) (Travis et al., 2013), which categorizes activity as mild (1–3 points), moderate (4–6 points), or severe (7–8 points). The study revealed significant correlations between IFX response during the induction phase and gender, disease extent, and endoscopic index of severity. However, no significant correlation was observed between age and biochemical indicator (Table 1).

**TABLE 1:** General data and clinical characteristics of enrolled SUC patients.

Parameters	Response (*n* = 36)	Unresponse (*n* = 14)	*P*
Gender (male,%)	14 (38.9)	8 (57.1)	< 0.001
Age (years old)	54.61 ± 13.50	58.43 ± 15.08	0.544
Disease extent (n,%)			< 0.001
E1	8 (22.2)	0 (0.00)	
E2	6 (16.7)	3 (21.4)	
E3	22 (61.1)	11 (78.6)	
Endoscopic index of severity (UCEIS) (n,%)			< 0.001
Mild (1–3)	0.00	0.00	
Moderate (4–6)	8 (22.2)	4 (28.6)	
Severe (7–8)	28 (77.8)	10 (71.4)	
CRP[IQRs], (mg/l)	23.92 [8.29.45.95]	43.57 [23.58,68.75]	0.183
ESR, (mm/h)	42.61 ± 30.82	62.00 ± 22.98	0.147
PLT, (×10^9^/L)	284.11 ± 108.25	312.43 ± 102.69	0.558
Hb[IQRs], (g/L)	102.00 [94.75, 119.75]	108.00 [97.00, 120.00]	0.671
Alb[IQRs], (g/L)	33.10 [29.73, 39.40]	31.80 [28.50, 32.70]	0.396

### 4.2 Fecal and mucosal microbiota analysis in different IFX induced outcome groups

**FIGURE 1:**
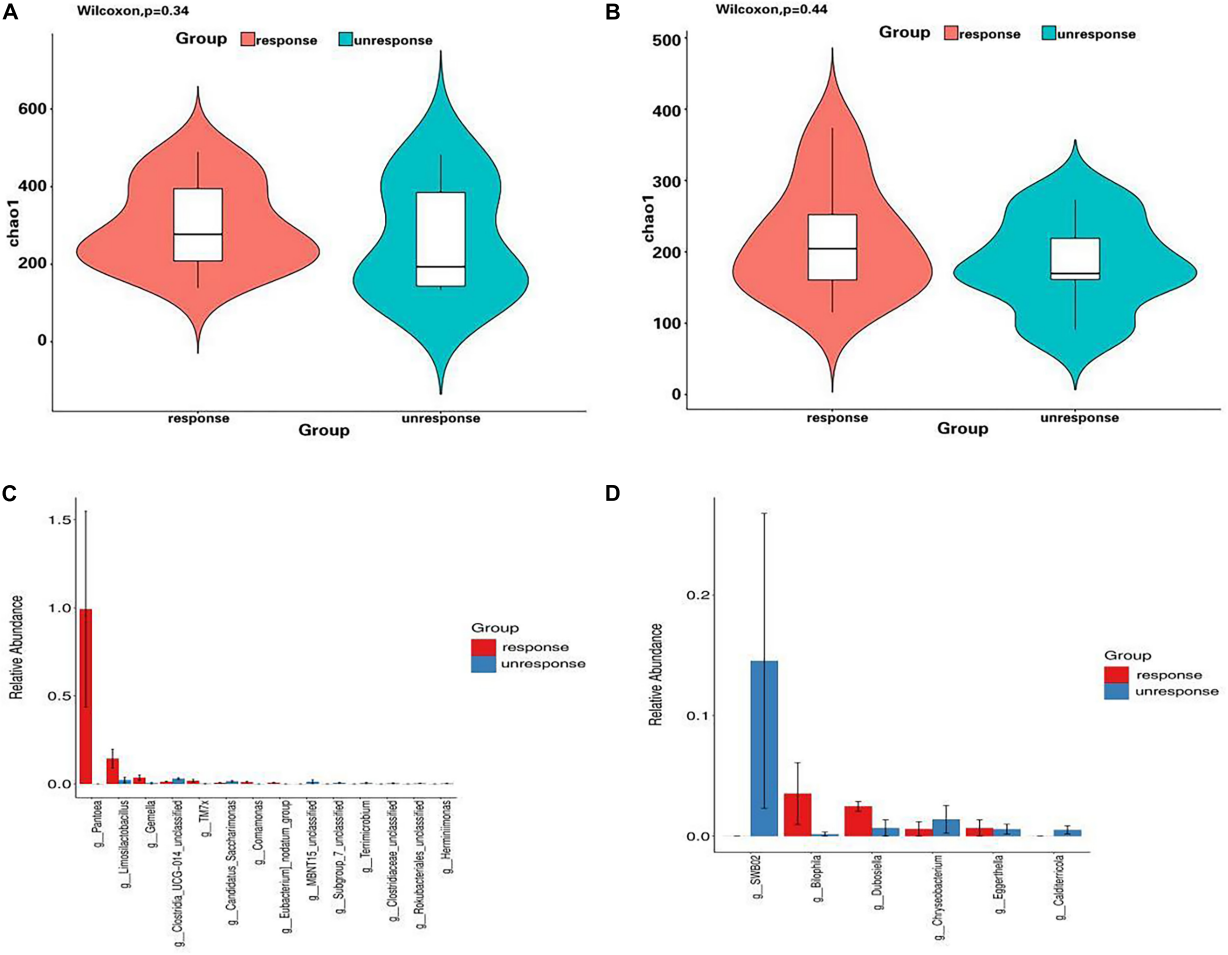
Differential bacterial analysis between different IFX induced outcome groups. **(A)** Alpha diversity analysis of fecal microbiota in response and unresponse group; **(B)** alpha diversity analysis of mucosal microbiota in response and unresponse group; **(C)** bar plot of significant bacterial differences at the genus level in fecal microbiota between response and unresponse group; **(D)** bar plot of significant bacterial differences at the genus level in mucosal microbiota between response and unresponse group.

After applying the aforementioned methods for differential bacteria identification, we observed that the alpha diversity of fecal and mucosal microbiota in the non-responder group did not exhibit a statistically significant difference compared to the responder group; however, a declining trend was evident (Figures 1A,B). There were differential bacteria expressions between the different IFX induced outcome groups (response or unresponse).

At the genus level, there were 14 significantly different bacterial species in feces and 6 significantly different bacterial species in mucosa between the two groups (Figures 1C,D).

### 4.3 The gut microbiota may serve as potential biomarkers for predicting the efficacy of IFX induction period in patients with SUC

**FIGURE 2:**
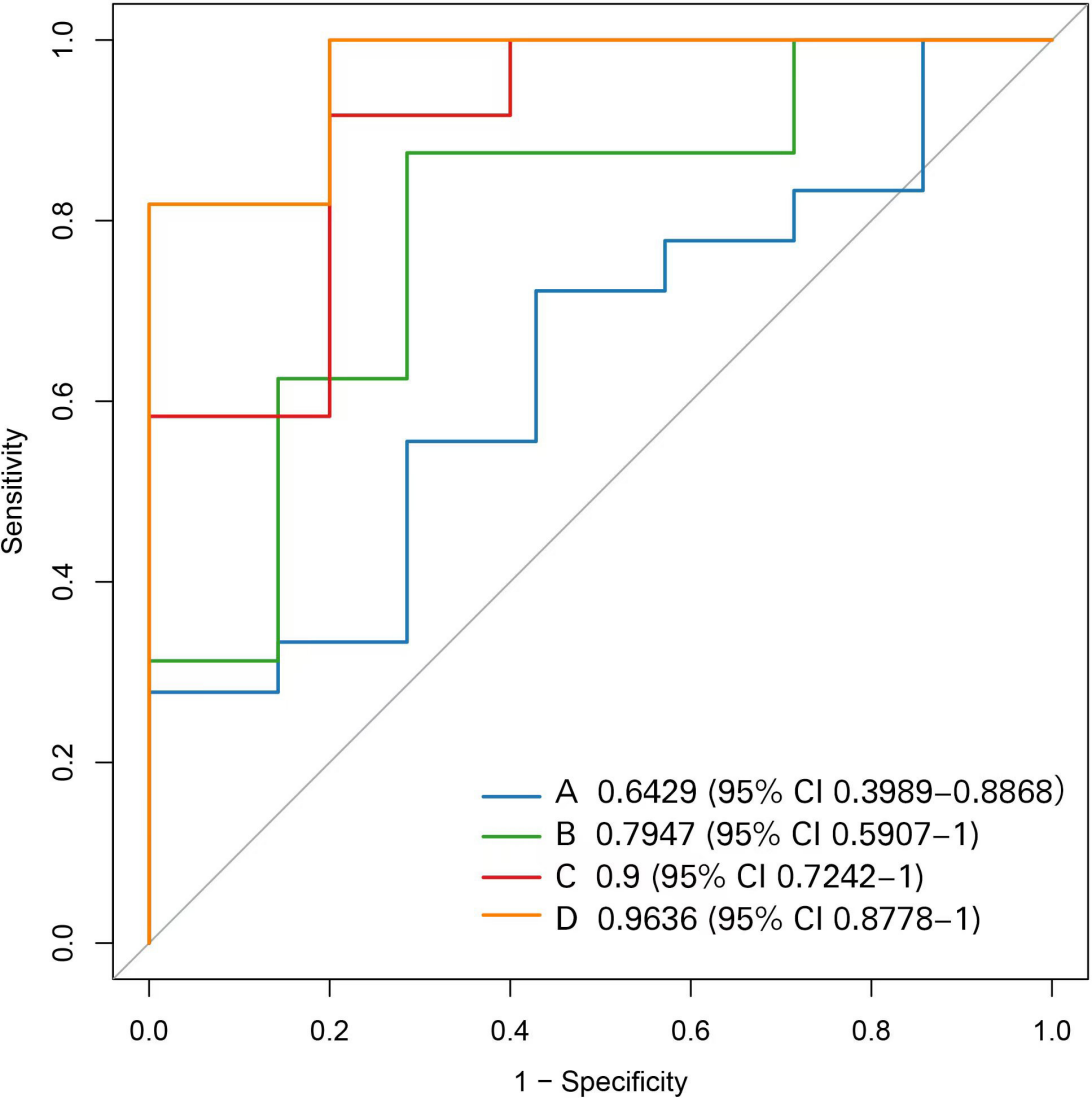
Receiver operating characteristic (ROC) curves depicting the AUC for predicting treatment response at week 14 in patients with SUC treated with IFX, utilizing various RF models. **(A)** Clinical data model; **(B)** clinical data and fecal microbiota model; **(C)** clinical data and mucosal microbiota model; **(D)** combined model incorporating clinical data, fecal microbiota, and mucosal microbiota.

To further test whether the gut microbiota provides biomarkers for prognosis of IFX induced treatment for SUC patients, we developed and assessed a predictive model based on baseline gut microbiota data (at week 0) to predict the IFX-induced outcome (response or unresponse) at week 14. The combination use of clinical data with fecal microbiota or mucosal microbiota, respectively, increased the prediction accuracy to 0.7947 and 0.9, respectively, compared to an accuracy of 0.6429 when relying solely on clinical data. Furthermore, the combined model incorporating clinical data, fecal microbiota, and mucosal microbiota can further improve the accuracy of prediction of the prognosis (to 0.9636) (Figure 2).

In RF model, the higher the Mean Decrease Gini (MDG) of a parameter, the more important it is. The integrated model utilizing clinical data, fecal microbiota, and mucosal microbiota emerged as the most effective predictive model for the 14-week clinical response to IFX treatment. The MDG values of various parameters in the model are presented in Table 2, indicating that PLT, ESR, *Dubosiella* in the mucosa, and *Gemella* in the feces were significant contributors to this model (Table 2). These findings underscore the potential of gut-microbiota signatures to stratify SUC patients and guide individualized therapy for improved IFX induced outcomes, yet they require validation in larger, independent cohorts.

**TABLE 2:** Mean decrease gini (MDG) values of features in the optimal predictive model.

Predictive factors	MDG
PLT	0.652211505
M_g_*Dubosiella*	0.607162926
ESR	0.575986808
F_g_*Gemella*	0.553336036
F_g_*Limosilactobacillus*	0.455684898
ALB	0.364102703
Age	0.339669564
F_g_ *Clostridia_ UCG-014_ unclassified*	0.333193701
M_g_ *Calditerricola*	0.306453286
F_g_ *Comamonas*	0.299845155
CRP	0.281919725
M_g_*Bilophila*	0.228732756
M_g_ *Chryseobacterium*	0.228062204
M_g_*SWB02*	0.227533122
HB	0.171886663
F_g_*Eubacterium*]_ *nodatum_group*	0.155930014
F_g_*Pantoea*	0.146130053
F_g_ *Candidatus_ Saccharimonas*	0.119468515
Gender	0.098270058
F_g_*Subgroup_ 7_ unclassified*	0.074103358
F_g_*TM7x*	0.055823521
Disease extent	0.051766378
F_g_*MBNT15_ unclassified*	0.044161538
M_g_*Eggerthella*	0.039327417
Endoscopic Index of Severity	0.012571429

MDG, Gini coefficient; F, Fecal microbiota; M, Mucosal microbiota; g, Genus level.

## 5 Metabolomic characteristics of fecal and mucosal samples in different IFX induced outcome groups

**FIGURE 3:**
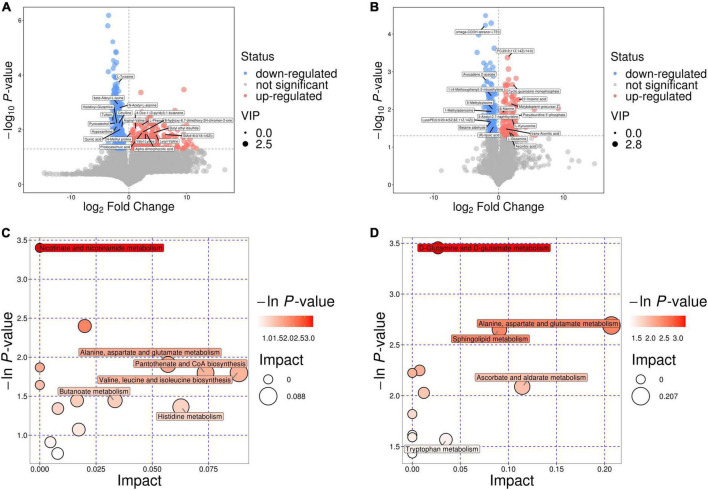
Metabolic differences between different IFX induced outcome groups in SUC. **(A)** Volcano plot of differential metabolites in fecal samples. **(B)** Volcano plot of differential metabolites in mucosal samples. **(C)** Bubble plot of enriched metabolic pathways in fecal samples. **(D)** Bubble plot of enriched metabolic pathways in mucosal samples..

By utilizing the aforementioned methods to classify patients according to their clinical response to IFX treatment for SUC, we identified significant differences in metabolite profiles between the two groups, as evidenced by both fecal and mucosal samples. Specifically, a total of 689 differential metabolites were identified in fecal samples. Among these metabolites were DL-phenylalanine, hydroxyisocaproic acid, L-tyrosine, and protocatechuic acid, among others. L-tyrosine, DL-phenylalanine, hydroxyisocaproic acid, and other compounds were observed at significantly higher relative concentrations in the non-responder group (Figure 3A). In the mucosal samples, a total of 377 differential metabolites were identified, including β-alanine, L-kynurenine, L-glutamine, sphingosine, and 1-methyladenosine, among others.

1-methyladenosine and other compounds were present at significantly higher relative levels in the non-responder group (Figure 3B). The most significantly enriched differential metabolic pathways in fecal samples between the two groups encompassed nicotinate and nicotinamide metabolism, butyrate metabolism, the biosynthesis of valine, leucine, and isoleucine, pantothenate and coenzyme A (CoA) biosynthesis, histidine metabolism, and the metabolism of alanine, aspartate, and glutamate (Figure 3C). The most significantly enriched differential metabolic pathways identified in mucosal samples encompassed alanine, aspartate, and glutamate metabolism; sphingolipid metabolism; ascorbate and aldarate metabolism; tryptophan metabolism; and D-glutamine and D-glutamate metabolism (Figure 3D). The shared metabolic pathway enriched in both fecal and mucosal samples was alanine, aspartate, and glutamate metabolism. This pathway presents a promising target for future research aimed at improving the efficacy of IFX treatment in patients with SUC.

## 6 Discussion

UC is a chronic, immune-mediated gastrointestinal inflammatory disorder of the gastrointestinal tract that, together with CD, constitutes the group of diseases known as IBD and exhibits significant heterogeneity. In recent years, biologics have increasingly become the primary therapeutic agents for UC (Siegel et al., 2020). Several authoritative guidelines recommend IFX as a first-line therapeutic option for patients with SUC (Rubin et al., 2019; Raine et al., 2022). However, IFX exhibits a ceiling effect, with an estimated 13–46% of IBD patients failing to respond or losing response within the first 12 weeks of treatment (Kopylov and Seidman, 2016). Notably, primary non-response—defined as a lack of clinical improvement following IFX induction therapy—highlights the urgent need for predictive tools that can support the implementation of more individualized treatment strategies (Gerich and McGovern, 2014). Currently, there is a lack of validated predictive models capable of accurately forecasting the efficacy of IFX in the treatment of SUC in real-world clinical practice. The aim of this study is to develop a personalized clinical decision-making model that can assist healthcare providers in improving the accuracy of treatment response prediction, reducing unnecessary healthcare expenditures for patients, and alleviating the broader society.

Several biochemical indices—PLT, CRP, ESR, and Hb—correlate with UC severity. Endoscopic parameters, including disease extent and the UCEIS, further inform prognosis (Chen et al., 2020). Nevertheless, we observed that these clinical variables alone were insufficient to predict IFX response at week 14 (AUC = 0.6429). We therefore identified the ten most differentially abundant bacterial taxa in fecal and mucosal samples between responders and non-responders and integrated them with clinical data to construct three random-forest models: (1) clinical variables plus fecal microbiota, (2) clinical variables plus mucosal microbiota, and (3) clinical variables combined with both fecal and mucosal microbiota. All three models predicted IFX response at week 14 (AUC = 0.795, 0.900, and 0.964, respectively), indicating that gut microbiota can serve as a biomarker of IFX induction efficacy in SUC, a conclusion that aligns with prior research on fecal microbiota in IBD (Boon et al., 2015; Plevris and Lees, 2022). This study demonstrates that mucosal microbiota combined with clinical parameters outperforms fecal microbiota in predicting 14-week clinical response to IFX in SUC, with the three-dimensional integration model (clinical parameters, fecal microbiota and mucosal microbiota) exhibiting optimal predictive performance. This finding aligns with contemporary perspectives in IBD microbiome research, which posit that mucosa-associated microbiota more directly reflects the pathophysiological state of the intestinal inflammatory microenvironment compared to fecal microbiota (Świrkosz et al., 2023).

In the pathogenesis of UC, compromised intestinal barrier function facilitates enhanced contact between mucosal microbiota and epithelial cells as well as lamina propria immune cells. Microbe-associated molecular patterns (MAMPs) activate NF-κB and MAPK signaling pathways via Toll-like receptors (TLRs) and NOD-like receptors, driving excessive production of pro-inflammatory cytokines (TNF-α, IL-6, IL-17) (Świrkosz et al., 2023). Consequently, mucosal microbiota composition not only reflects instantaneous disease activity but also harbors predictive potential for anti-TNF-α therapeutic response. Conversely, although fecal microbiota offers the advantage of non-invasive collection, it predominantly represents luminal microbial communities and may fail to accurately capture key microbial signatures directly interacting with mucosal immunity (Dutta et al., 2025).

Recent multi-cohort studies have established that baseline gut microbial features effectively predict biologic treatment outcomes in IBD. Zheng et al. integrated data from 231 IBD patients receiving biologics (including IFX, adalimumab, and ustekinumab), demonstrating significantly elevated α-diversity in responders at baseline. They constructed a predictive model based on 10 key genera, achieving AUCs of 0.895 in the training cohort and 0.750 in the validation cohort. Notably, these predictive genera predominantly belong to the class *Clostridia*, possessing short-chain fatty acid (SCFA)-producing capabilities critical for immune regulation and intestinal barrier maintenance (Zheng et al., 2025). The superiority of mucosal microbiota observed in our study likely stems from its more direct representation of these functional taxa interacting with the host inflammatory microenvironment.

From a multi-omics integration perspective, Lee et al. demonstrated that models combining clinical, metagenomic, metabolomic, and proteomic data achieved AUCs of 0.963 for predicting anti-cytokine therapy (anti-TNF-α and anti-IL-12/23) response, significantly outperforming single clinical feature models (AUC 0.624) (Chen et al., 2025b). Our three-dimensional integration strategy employing clinical parameters, fecal microbiota, and mucosal microbiota embodies the precision medicine paradigm shift from single-omics to multi-dimensional biomarker systems. Fecal microbiota provides a non-invasive overview of the gut microbial ecosystem, whereas mucosal microbiota offers disease-site-specific microenvironmental information; their complementary integration enables construction of a more comprehensive predictive framework.

Further untargeted metabolomic profiling revealed significant metabolite disparities between infliximab (IFX) responders and non-responders in both fecal and mucosal compartments, with a higher number of differential metabolites detected in feces. These findings suggest that fecal metabolic activity is more pronounced and complex in patients with SUC. Pathway enrichment analysis showed that fecal samples were characterized by significant enrichment of alanine, aspartate, and glutamate metabolism; valine, leucine, and isoleucine biosynthesis; pantothenate and CoA biosynthesis; histidine metabolism; and butyrate metabolism. In contrast, mucosal biopsies exhibited enrichment of alanine, aspartate, and glutamate metabolism; tryptophan metabolism; ascorbate and aldarate metabolism; threonine metabolism; and D-glutamine and D-glutamate metabolism.

Recent longitudinal cohort studies have identified that the therapeutic response to IFX is closely associated with the expression ratio of indoleamine 2,3-dioxygenase 1 (IDO1) to quinolinate phosphoribosyltransferase (QPRT) in colonic mucosa (Welz et al., 2024). Responders exhibited characteristic alterations in the kynurenine pathway during the early phase of treatment, suggesting that the baseline status of this pathway may influence the sensitivity to anti-TNF therapy. This finding is highly consistent with the observed differences in tryptophan metabolism identified in our study.

Our study identified alanine, aspartate, and glutamate metabolism as the sole pathway significantly enriched across both sample types. The gut microbiota can metabolize glutamate into γ-aminobutyric acid (GABA), thereby alleviating intestinal barrier dysfunction via activation of GABA_A receptors or inhibition of the AMPK pathway (Niu et al., 2025). Furthermore, aspartate can be converted into glutamate via transamination, with the latter serving as an indispensable precursor for glutathione synthesis (a critical antioxidant), thereby modulating the oxidative stress status of intestinal epithelial cells (Chen et al., 2025a). This metabolic pathway exhibits significant dysregulation across multiple sample types in IBD patients (colonic mucosa, feces, and blood), representing one of the most consistent alterations in the IBD metabolomic signature (Liu et al., 2024). Alanine, aspartate, and glutamate serve as critical energy metabolic substrates that enter the tricarboxylic acid (TCA) cycle through conversion to pyruvate, oxaloacetate, and α-ketoglutarate, respectively, thereby providing ATP for colonic epithelial cell proliferation, differentiation, and intestinal barrier maintenance (Liu et al., 2024). UC patients frequently manifest energy metabolic disturbances, characterized by reduced levels of TCA cycle intermediates (e.g., α-ketoglutarate, succinate, fumarate), indicative of mitochondrial dysfunction and insufficient energy supply (Liu et al., 2024). As an anti-TNF-α antibody, IFX attenuates inflammation by neutralizing pro-inflammatory cytokines, and this inflammatory resolution may be accompanied by restoration of mucosal energy metabolic status. Responders to therapy may possess inherently superior energy metabolic capacity at baseline, or effectively reverse metabolic disturbances through IFX treatment.

The key amino acids in this pathway exert direct immunomodulatory functions. Glutamate and glutamine regulate mTOR signaling, NF-κB activity, and macrophage polarization, thereby suppressing pro-inflammatory cytokine (TNF-α, IL-1β, IL-6) release and promoting M2 anti-inflammatory macrophage differentiation (Zheng et al., 2023). In IBD, glutamine promotes intestinal epithelial cell proliferation via MAPK pathway activation, enhances tight junction protein expression to maintain mucosal barrier integrity, and concurrently reduces IFN-γ and TNF-α production through inhibition of NF-κB and STAT signaling pathways (Zheng et al., 2023). Recent studies have further demonstrated that correction of glutamate metabolic dysregulation can suppress NF-κB signaling and downstream COX-2 expression, ameliorating intestinal inflammation (Jian et al., 2025).

The gut microbiota and host jointly participate in amino acid metabolism. Multiple studies have shown that fecal glutamate levels are significantly elevated in UC, whereas serum/plasma glutamine levels are decreased (Niu et al., 2025). This discrepancy likely reflects competitive alterations in amino acid utilization between the host and microbiota under inflammatory conditions. SCFA-producing taxa (e.g., *Faecalibacterium*, *Roseburia*) are intimately associated with amino acid metabolism, and their abundance fluctuations may modulate metabolic flux through the glutamine-glutamate-α-ketoglutarate axis (Ventin-Holmberg et al., 2025). IFX responders typically exhibit restoration of microbial community structure, particularly increased butyrate-producing bacteria, which may through microbe-host metabolic interactions, establishing a positive feedback loop (Ventin-Holmberg et al., 2025).

Our findings may serve as a valuable reference for future targeted metabolic investigations, aiding in the identification of metabolites capable of more precisely predicting IFX efficacy in severe ulcerative colitis. Clinically, these insights could be translated into simplified diagnostic panels utilizing quantitative PCR or targeted metabolomics to enhance accessibility and enable practical implementation in routine IBD practice.

Currently, therapeutic decision-making in IBD relies predominantly on clinical symptoms and inflammatory biomarkers, yet lacks reliable tools for predicting individualized treatment responses. Prediction models incorporating mucosal microbiota and specific metabolic pathways enable pre-treatment patient stratification, identifying high-probability responders likely to benefit from IFX therapy while prompting consideration of alternative therapeutic options (e.g., ustekinumab, vedolizumab, or JAK inhibitors) for low-probability responders, thereby averting delays in effective treatment and mitigating unnecessary healthcare resource utilization.

Although mucosal sample acquisition necessitates colonoscopy and carries inherent invasiveness, baseline endoscopic evaluation constitutes a standard component of the diagnostic and therapeutic workflow for SUC; consequently, collection of mucosal biopsies remains clinically feasible and operationally practical in this patient population. Integration of microbiome analysis into routine histopathological workflows, combined with predictive algorithms incorporating clinical parameters, holds substantial promise as an adjunctive decision-support tool for guiding therapeutic selection.

Nonetheless, this study is subject to certain limitations. First, the stringent exclusion criteria—specifically the omission of patients previously treated with antibiotics, mesalazine, biologics, corticosteroids, or immunosuppressive agents—while necessary to minimize confounding medication effects on gut microbiota and metabolites, resulted in a relatively small sample size (*n* = 50). This constraint limits the generalizability of our findings, and the high AUC (0.9636) may reflect overfitting to this specific cohort despite our cross-validation safeguards. Second, the lack of an external validation cohort precludes rigorous assessment of model transportability to independent populations. Third, the clinical response was assessed solely at week 14, and treatment efficacy was categorized based on this observation. This approach may have overlooked potential delayed responses.

We plan to expand the sample size through multi-center enrollment and externally validate the model using independent cohorts. By integrating clinical, microbiome, and metabolomic data, we aim to construct multi-dimensional predictive models for 14-week IFX response in SUC, ultimately translating these into clinically applicable decision support tools to advance precision medicine in IBD.

## Data Availability

The data presented in the study are deposited in the NCBI repository, accession number PRJNA1465084.

## Footnotes

1
http://www.genome.jp/kegg/

2
http://www.metaboanalyst.ca/
